# MATLAB Algorithms for Diameter Measurements of Textile Yarns and Fibers through Image Processing Techniques

**DOI:** 10.3390/ma15041299

**Published:** 2022-02-10

**Authors:** Mohamed Abdelkader

**Affiliations:** Department of Advanced Materials, Institute for Nanomaterials, Advanced Technologies and Innovation, Technical University of Liberec, 46117 Liberec, Czech Republic; mohamed.fawzy@mena.vt.edu

**Keywords:** yarn, yarn’s helix model, fiber, diameter, hairiness, image processing, MATLAB, Hough transform

## Abstract

Textile yarns are the fundamental building blocks in the fabric industry. The measurement of the diameter of the yarn textile and fibers is crucial in textile engineering as the diameter size and distribution can affect the yarn’s properties, and image processing can provide automatic techniques for faster and more accurate determination of the diameters. In this paper, facile and new methods to measure the yarn’s diameter and its individual fibers diameter based on image processing algorithms that can be applied to microscopic digital images. Image preprocessing such as binarization and morphological operations on the yarn image were used to measure the diameter automatically and accurately compared to the manual measuring using ImageJ software. In addition to the image preprocessing, the circular Hough transform was used to measure the diameter of the individual fibers in a yarn’s cross-section and count the number of fibers. The algorithms were built and deployed in a MATLAB (R2020b, The MathWorks, Inc., Natick, Massachusetts, United States) environment. The proposed methods showed a reliable, fast, and accurate measurement compared to other different image measuring softwares, such as ImageJ.

## 1. Introduction

The textile yarn is a group of fibers twisted together [1], and the diameters of the yarn and its fibers are important characteristics in the textile industry. Yarn diameter is a fundamental parameter of a textile yarn that is used for calculations of yarn different parameters such as twist angle which affects many yarn properties, such as yarn’s strength [2], hairiness, and appearance [3]. The fiber diameter can affect the yarn’s porosity and its mechanical properties [4]. In the literature, the measuring methods mainly address the determination of yarn’s diameter, there are different techniques to measure the yarn’s diameter including capacitive, optical, mechanical, and electromechanical measuring techniques [5]. The previous methods require special tools or preparations which can be financially challenging, hence having a simple and computerized method would be a suitable solution to measure the yarn diameter efficiently.

On the other hand, image processing has been extensively used for microscopic images analysis, and it can be used for morphology characterization and appearance evaluation [6]. The microscopic images are stored on computers in several digitized formats [7], where these images can be easily processed with images processing algorithms [8]. There are different applications for image processing to obtain the desired information, and the preference of using one method or another can be related to the processing speed and/or the available computer’s memory. Therefore, digital image processing found multiple applications, such as the reconstruction of microscopic images [9], the characterization of filtration membranes [10], and the analysis of porosity in cementitious materials [11]. The Wang’s group proposed an image processing-based technique to evaluate the yarn evenness and hairiness [12]. Manal’s group used the image processing and machine learning to determine the characteristics such as yarn tenacity, elongation%, and coefficient of mass variation% of the cotton’s yarn [13]. From these examples and many more in the different research fields, image processing techniques possess considerable impact to achieve automatic and accurate analysis.

Exploring the literature research on the problem of measuring the yarn diameter using image processing techniques, a group of researchers used spatial methods [14] to analyze the yarn’s core and a technique in the frequency domain based on Fourier transform and filtration. Both approaches were successful in determining the twist angle of yarns which can be used to determine the yarn’s diameter. The commercial system USTER (USTER TECHNOLOGIES, Uster, Switzerland) [15] is used to characterize the yarn, such as its diameter and hairiness. This tool can be an expensive system that can limit some researchers to characterize their yarns fast and cost-effectively; hence, using the proposed image processing techniques can be a reasonable and effective alternative to characterize the yarns and their individual fibers.

In this paper, a new and facile method is introduced to measure the yarn diameter and individual fibers count and diameter. The method of measuring the diameter of the yarn is based on finding the yarn’s body, binarizing it, filling in-between voids, and removing the hairiness of the yarn. The other algorithm measures the diameter of the individual fibers of a cross-sectional image of the yarn, the detection of the circular or circular-like cross-sections of fibers is based on the circular Hough transform [16] that can find and characterize circular or near-circular shapes in an image. Both algorithms are deployed in a MATLAB environment.

## 2. Materials and Methods

### 2.1. The Measurement of the Yarn’s Diameter

#### The Yarn’s Helix Model

**Figure 1:**
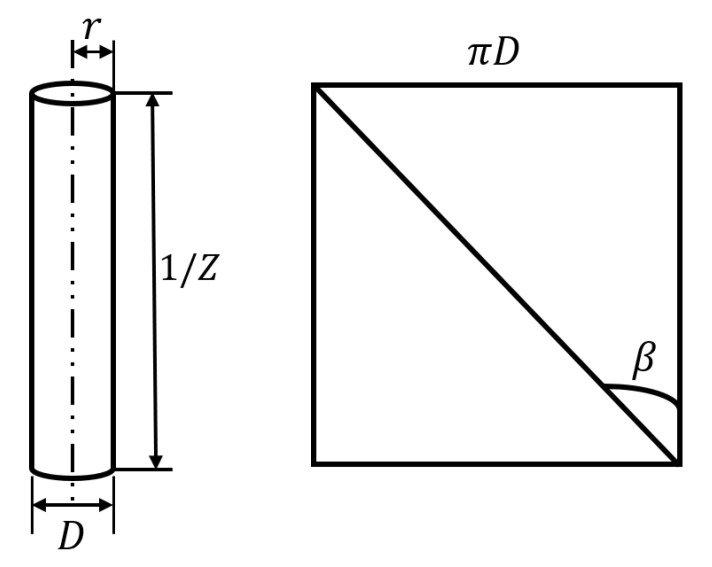
Yarn helix model (where: r is the distance of the fiber from the yarn’s helix center, D is the yarn’s diameter, Z is the twist number, and β is the twist angle), inspired by [17].

The theoretical yarn twist can be estimated based on different models of the yarns. One of the simplest models that represent the textile yarn is the helix model, which assumes that all fibers are identical and have the same cross-section. The helix model is an idealized mathematical model for textile yarns [17], and Figure 1 shows that model where the bending line within the cylinder (yarn body) represents the fiber.

The rectangle in Figure 1 is the unfolded version of the yarn that appears as a cylinder at the left of Figure 1. By assuming the fiber location on the yarn’s surface and having a yarn diameter D, the distance of the fiber from the yarn’s axis will be r = D/2. Then, we can calculate the diameter of the yarn using the relationship:

(1) $$\tan\left(\beta \right)=\frac{2\pi r}{1/Z}=\pi \mathrm{DZ}$$

(2) $$∴D=\frac{\tan\left(\beta \right)}{\pi Z}$$

Equation (2) shows a theoretical calculation of the yarn’s diameter that depends on several yarn parameters such as the twist numbers and the twist angle. In the proposed algorithm an accurate measurement of the real images is proposed, these images can be for example optical microscopy images and scanning electron microscopy images. In this study, yarn images were obtained using a Zeiss Ultra Plus field emission scanning electron microscope, UHR FE-SEM Carl Zeiss Ultra Plus (Zeiss, Jena, Germany). SEM images were obtained at an accelerating voltage of 5 kV at a magnification level of 50x, the yarn samples were cut, spattered, and placed to the SEM holder in the ambient room conditions

### 2.2. The Algorithm of Yarn’s Diameter

**Figure 2:**
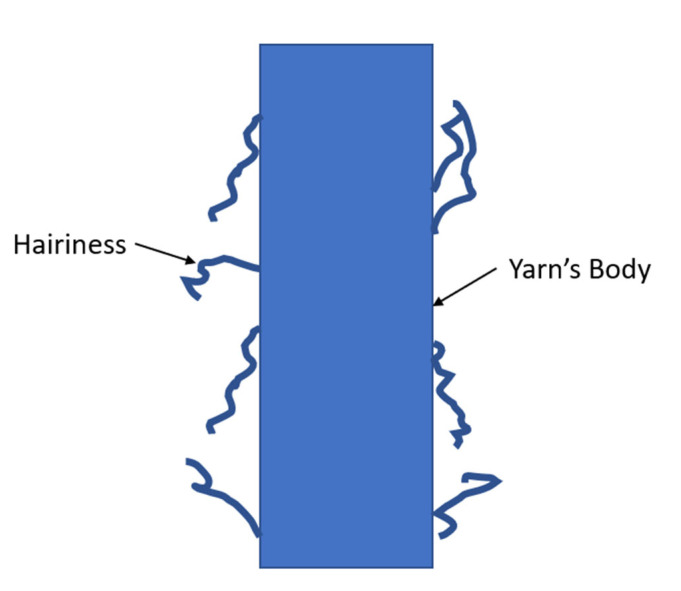
Illustration of the yarn’s hairiness.

The MATLAB algorithm starts by allowing the user to select the targeted yarn image, then the user makes a cropped part of the image that contains the full body of the yarn. After that, the user measures the scale par in pixels through a built-in tool in MATLAB and enters the actual scale bar length in micrometers, this is necessary to calculate the actual diameters in mm by applying the direct substitution explained in Equation 3 inside the algorithm Once the user has inserted the scale bar into the algorithm, the algorithm starts to preprocess the image, thresholding the image, binarizing the image, and filling the voids in the binarized body of the yarn. The algorithm tries to minimize the hairiness of the yarn as much as possible, when the sum of hairiness pixels is less than a third of diameter, the hairiness of the yarn is eliminated. Figure 2 illustrates the hairiness of the fiber which the algorithm eliminates during the calculations.

(3) $$\mathrm{Diametr}\;\mathrm{in}\;\mathrm{mm}=\frac{\mathrm{Measured}\;\mathrm{bar}\;\mathrm{length}\;\mathrm{in}\;\mathrm{pixels}\;\times \;\mathrm{Bar}\;\mathrm{length}\;\mathrm{in}\;µm\;\left(\mathrm{from}\;\mathrm{the}\;\mathrm{microscpic}\;\mathrm{image}\right)}{\mathrm{Bar}\;\mathrm{length}\;\mathrm{in}\;\mathrm{pixels}\;\times 1000}$$

**Figure 3:**
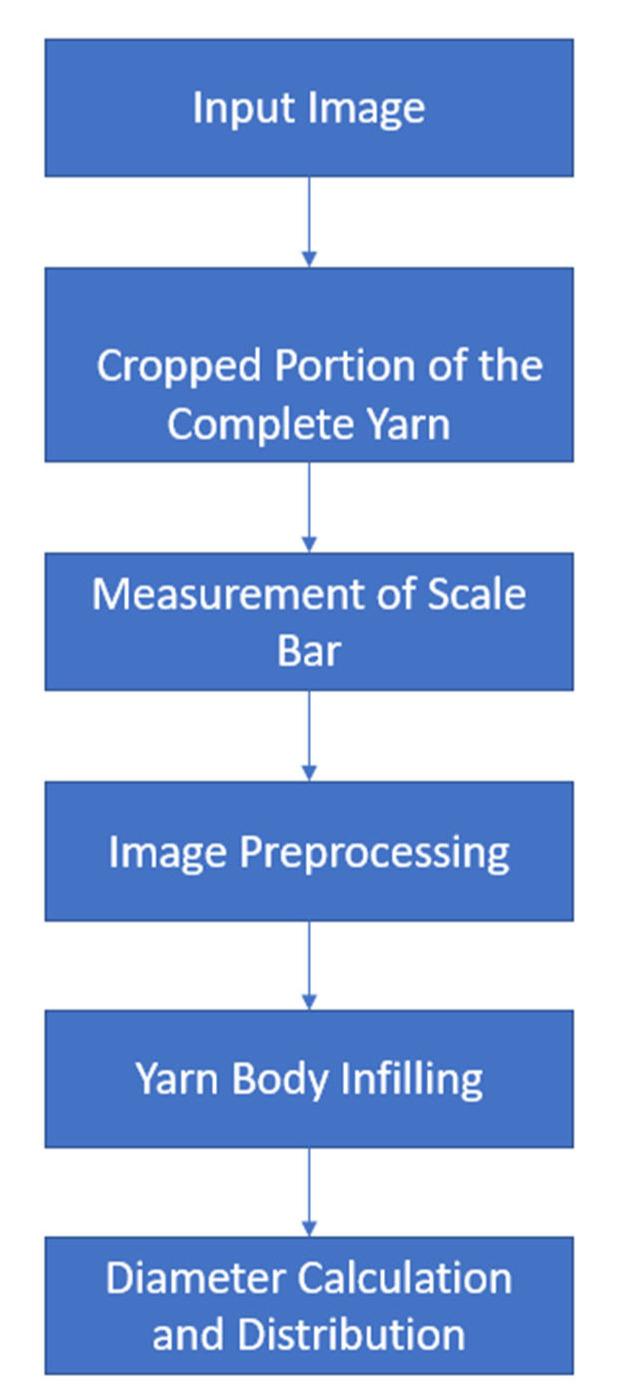
Summary of yarn’s diameter algorithm.

The diameter is calculated by summing the pixels across each row of the image after eliminating the hairiness of the yarn (assuming that body of the yarn is perpendicular on the x-axis). Then, the algorithm calculates the average yarn diameter in mm and the distribution of the diameter over the yarn’s body down to a resolution of one pixel. Figure 3 summarizes the overall algorithm to calculate the yarn’s diameter.

### 2.3. The Measurement of the Fibers’ Diameter

#### 2.3.1. Obtaining Yarn’s Cross Sections

The cross-sectional images of the yarn can be obtained by slicing the yarn using a microtome and then imaging these slices using an optical microscope [18,19]. In addition to the microtome approach, the yarn’s slices can be obtained by micro-computed tomography scanning machines, where the yarn is three-dimensionally (3D) scanned and a digital actual 3D model of the yarn is constructed [20,21,22]. The cross-section images/slices can be reconstructed from that scan, the computed tomography slices give insight into the internal and external structure of the yarn; hence, these slices can be useful for modeling and understanding the parameters of the textile yarn. In this study, the micro-CT scanner Rigaku nano3DX (Rigaku Corporation, Tokyo, Japan) was used with tube voltage in range from 20 to 50 kV and current up to 30 mA with a voxel resolution around 0.5 µm to scan the yarn and obtain the cross-section images.

#### 2.3.2. The Algorithm of the Yarn’s Diameter

**Figure 4:**
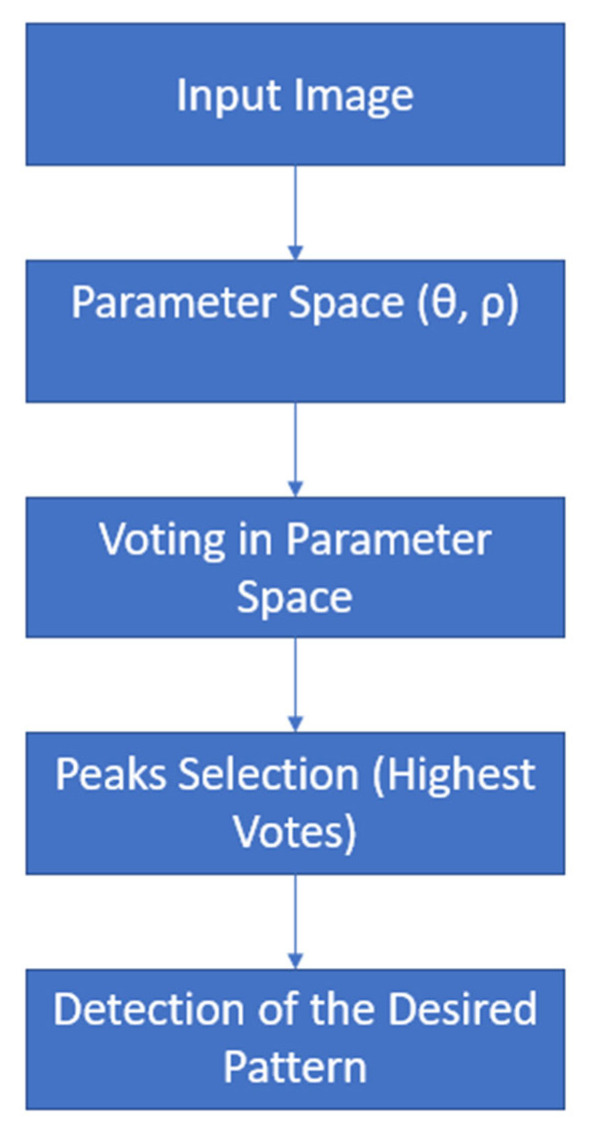
Summary of the yarn’s diameter algorithm.

The detection of the yarn’s diameter is based on the detection and measurement of circular shapes/ circular alike shapes, this is done by using the Hough transform. The Hough transform was initially invented as an electrical circuit to identify and recognize the pattern of straight lines [23]. The Hough transform is a powerful tool in image processing as it can detect basic shapes such as lines and more general patterns (such as circles or ellipses) [24]. The Hough transform is mainly applied to binary images, and, to simplify the problem of line recognition, each white pixel in a binary image with coordinates (x,y) is mapped into the parameter space (ρ,θ). Figure 4 illustrates the general working principle of the Hough transform.

The targeted pattern shape in the yarn’s diameter measurement problem is the circular shape; hence, the circular Hough transform is used to detect and measure the radius of circular/circular alike shapes in the images [16,25,26,27]. In this study, these circular shapes represent the cross-sections of individual fibers along the yarn.

**Figure 5:**
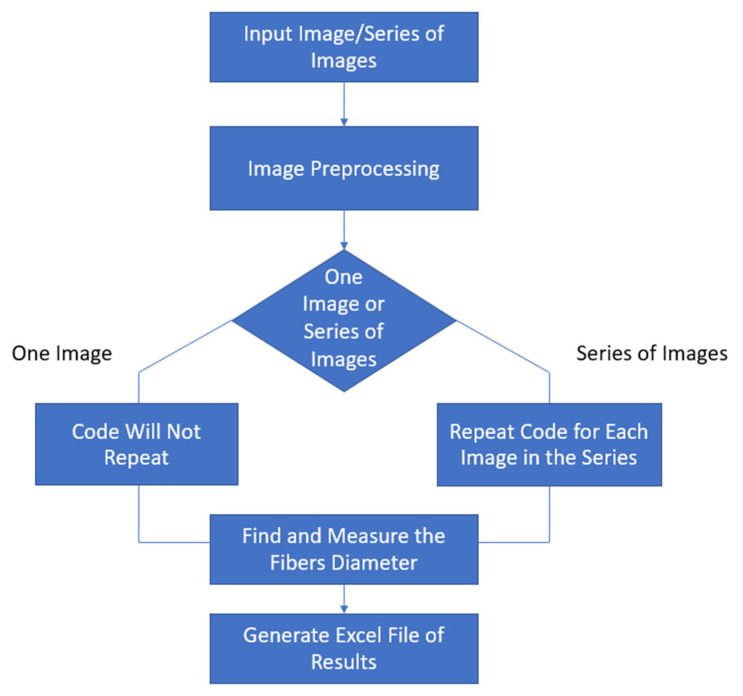
Summary of the fiber’s diameter algorithm.

The algorithm starts by reading the image or series of images at a certain path that the user chooses by creating a list of all images at the chosen path. The, the code loops and applies the algorithm on each single image in that list and if the path has more than one image, then the user measures any diameter of a single fiber that can give a certain range in which the Hough transform will find and measure the fibers. The algorithm then asks the user whether the user wants to process the chosen image or all the images in the specified path. The algorithm applies the transform, detects the fibers, and measures each fiber diameter, then an excel file is generated with the coordinates of each fiber and its diameter in pixels. Figure 5 summarizes the diameter of the fiber algorithm as shown below.

The open-source MATLAB codes for both algorithms can be accessed, downloaded, and edited from GitHub which is a platform for hosting and sharing the codes [28], allowing other researchers in the field to explore and enhance the provided codes.

## 3. Results and Discussion

### 3.1. Yarn’s Results

**Figure 6:**
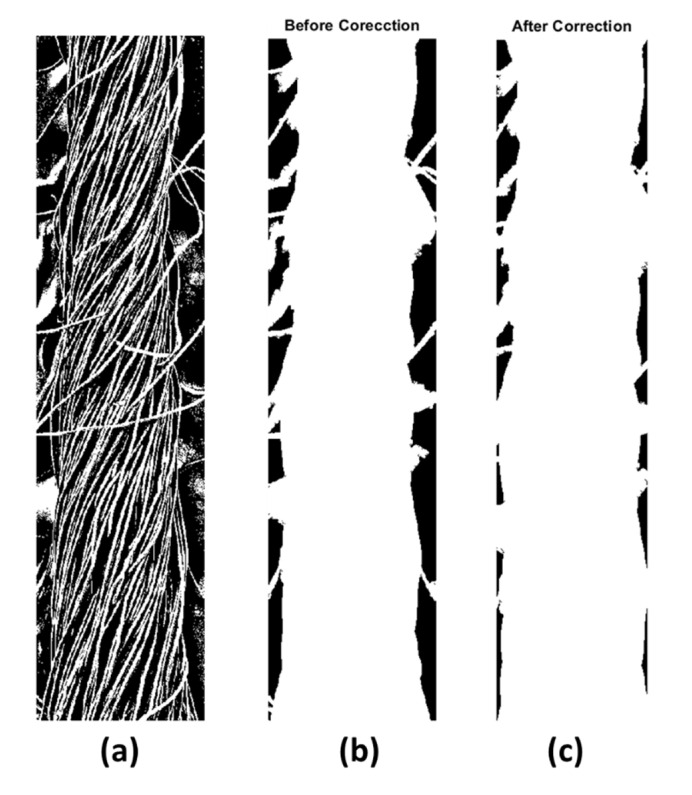
Processing of the cropped yarn body: (**a**) the binarized yarn body; (**b**) yarn body with hairiness; and (**c**) the corrected yarn body after removing hairiness.

After running the yarn’s diameter algorithm, the user selects the targeted image, crops the yarn body, measures the scale bar, and inters its value in micrometers. The algorithm displays the cropped and preprocessed yarn’s body, the body with excess hairiness, and the corrected yarn. Figure 6 shows the algorithm outputs of processing the yarn’s body.

**Figure 7:**
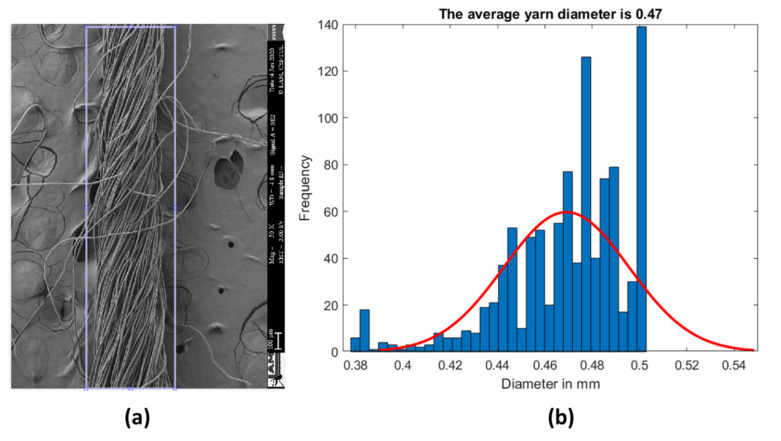
(**a**) The processed example SEM image with the selection of yarn’s body and (**b**) the diameter distribution showing the average diameter obtained by the proposed algorithm.

After that, the algorithm starts to calculate the diameter of the yarn across all the rows of the picture and finds the average diameter and its statistical distribution as shown in Figure 7. The processed image had a diameter of 0.47 ± 0.03 mm.

**Figure 8:**
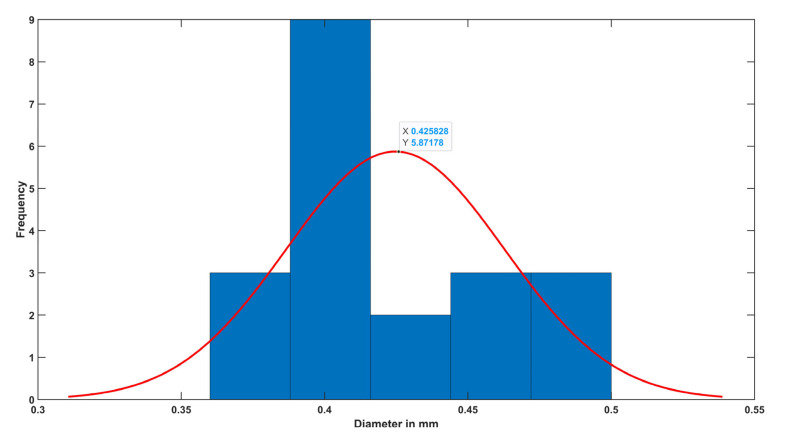
The diameter distribution showing the average diameter obtained manually by ImageJ.

In order to verify the efficacy of the proposed method, the yarn’s diameter was measured manually using ImageJ [29]. A number of 20 measurements were completed equally distributed across the yarn’s body. The diameter distribution in Figure 8 shows an average diameter of 0.42 ± 0.03 mm compared to 0.47 ± 0.03 mm that was obtained by the proposed algorithm.

### 3.2. Fiber Results

**Figure 9:**
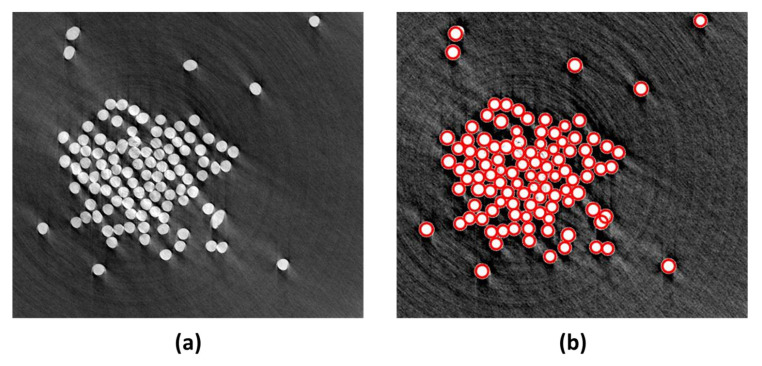
(**a**) The yarn cross-section before processing, and (**b**) the cross-section image after being processed by the algorithm.

**Figure 10:**
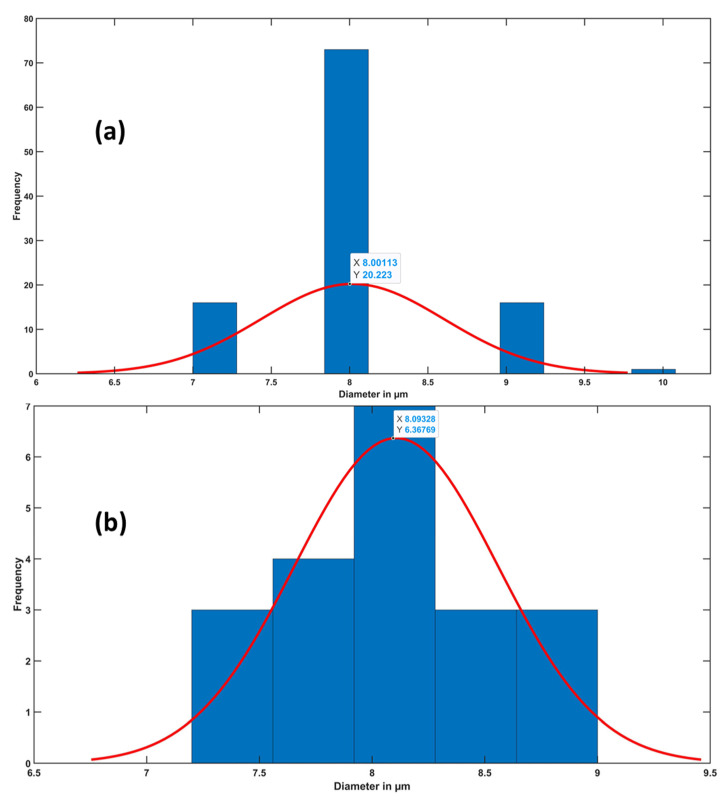
(**a**) The fibers’ diameters distribution of the cross-section image in Figure 9a obtained by the proposed algorithm, and (**b**) the fibers’ diameters distribution of the cross-section image in Figure 9a obtained manually by ImageJ.

After running the fiber’s diameter algorithm, the user selects the targeted thread, and the algorithm reads all the images in the thread. The images are listed in a list and the code is looped on each image if the thread has more than one image. The user measures any fiber diameter that will set a certain range of pixels that forms an average radius of the circular shapes that the algorithm finds through the Hough transform, Figure 9 shows the cross-section image of a yarn obtained from a CT scan before and after being processed with the algorithm. The generated excel file showed that the image contains 106 fibers with a mean diameter of 8.00 ± 0.64 µm as shown in Figure 10a. In order to verify the efficacy of the proposed method, the fibers’ diameters were measured manually using ImageJ. A number of 20 measurements were carried out across the cross-section image. The diameter distribution in Figure 10 shows an average diameter of a single fiber around 8.00 ± 0.64 µm obtained by the proposed algorithm compared to 8.06 ± 0.45 mm obtained by ImageJ.

## 4. Conclusions

Yarn diameter and its fiber diameters determination are crucial in the textile industry, and image processing can provide fast and accurate techniques that can measure these diameters fast and efficiently. The two proposed methods showed reliability and efficiency in calculating the diameters in an automated manner. The yarn’s diameter method successfully eliminated the hairiness of the yarn and gave an average diameter of 0.47 ± 0.03 mm after applying the algorithm on the sample image compared to 0.42 ± 0.03 mm obtained manually by ImageJ software. The fiber’s diameter method successfully counted all the fibers in the cross-section image and generated an excel file of that data showing an average diameter of 8.00 ± 0.64 µm obtained by the proposed algorithm compared to 8.06 ± 0.45 mm obtained by ImageJ. The proposed algorithms can be applied to a single image or a thread that contains a series of images, providing fast and automated image processing-based measuring technique compared to ImageJ to measure the diameters of textile yarns and fibers. The proposed methods for both measurements of the yarn’s diameter and individual fiber’s diameter can be further extended to other microscopic structures such as nanofibers and organisms such as cells and are not limited only to textile yarn microscopic images.

## Data Availability

Not applicable.
